# LP-925219 maximizes urinary glucose excretion in mice by inhibiting both renal SGLT1 and SGLT2

**DOI:** 10.1002/prp2.129

**Published:** 2015-03-31

**Authors:** David R Powell, Melinda G Smith, Deon D Doree, Angela L Harris, Wendy W Xiong, Faika Mseeh, Alan Wilson, Suma Gopinathan, Damaris Diaz, Nicole C Goodwin, Bryce Harrison, Eric Strobel, David B Rawlins, Ken Carson, Brian Zambrowicz, Zhi-Ming Ding

**Affiliations:** 1 Lexicon Pharmaceuticals Inc.The Woodlands, Texas; 2 Lexicon Pharmaceuticals Inc.Princeton, New Jersey

**Keywords:** Diabetes, knockout mice, SGLT1, SGLT2, small molecule, urinary glucose excretion

## Abstract

Sodium-glucose cotransporter 2 (SGLT2) inhibitors are a new class of oral anti-diabetic agents that improve glycemic control by inhibiting SGLT2-mediated renal glucose reabsorption. Currently available agents increase urinary glucose excretion (UGE) to <50% of maximal values because they do not inhibit SGLT1, which reabsorbs >50% of filtered glucose when SGLT2 is completely inhibited. This led us to test whether LP-925219, a small molecule dual SGLT1/SGLT2 inhibitor, increases UGE to maximal values in wild-type (WT) mice. We first tested LP-925219 inhibition of glucose transport by HEK293 cells expressing SGLT1 or SGLT2, and then characterized LP-925219 pharmacokinetics. We found that LP-925219 was a potent inhibitor of mouse SGLT1 (IC_50_ = 22.6 nmol/L) and SGLT2 (IC_50_ = 0.5 nmol/L), and that a 10 mg/kg oral dose was bioavailable (87%) with a long half-life (7 h). We next delivered LP-925219 by oral gavage to WT, SGLT1 knockout (KO), SGLT2 KO, and SGLT1/SGLT2 double KO (DKO) mice and measured their 24-h UGE. We found that, in vehicle-treated mice, DKO UGE was maximal and SGLT2 KO, SGLT1 KO, and WT UGEs were 30%, 2%, and 0.2% of maximal, respectively; we also found that LP-925219 dosed at 60 mg/kg twice daily increased UGE of SGLT1 KO, SGLT2 KO, and WT mice to DKO UGE levels. These findings show that orally available dual SGLT1/SGLT2 inhibitors can maximize 24-h UGE in mammals, and suggest that such agents merit further evaluation for their potential, in diabetic patients, to achieve better glycemic control than is achieved using selective SGLT2 inhibitors.

## Introduction

Sodium-glucose cotransporter 2 (SGLT2), the major renal glucose transporter, is responsible for reabsorbing 97–98% of glucose filtered by the kidney under normal physiologic conditions (Vallon et al. 2011; Wright et al. 2011; Powell et al. 2013a; Rieg et al. 2014). Individuals lacking functional SGLT2 have familial renal glucosuria (OMIM 182381), a benign condition associated with increased UGE but not with hypoglycemia or other health problems (Santer and Calado 2010; Wright et al. 2011). Sodium-glucose transporter 1 (SGLT1) is also expressed in the kidney, but further downstream in the proximal tubule than SGLT2; in this position, SGLT1 has the opportunity to reabsorb any filtered glucose not reabsorbed by SGLT2 (Wright et al. 2011; Powell et al. 2013a; Rieg et al. 2014). In contrast to this minor role in renal glucose reabsorption, SGLT1 is the major intestinal glucose transporter (Wright et al. 2011). Individuals lacking functional SGLT1 show little if any increase in UGE but can develop an intestinal malabsorption syndrome, glucose-galactose malabsorption (OMIM 182380), when they ingest a diet containing these sugars (Wright et al. 2011).

SGLT2 is the target of new orally available compounds designed to improve glycemic control in patients with type 2 diabetes (T2D). All of these compounds are highly selective inhibitors of SGLT2 compared to SGLT1 except for sotagliflozin, also known as LX4211, which is a dual inhibitor of SGLT2 in the kidney to increase UGE, and SGLT1 in the intestine to delay glucose absorption (Chao and Henry 2010; Abdul-Ghani et al. 2012; Musso et al. 2011; Zambrowicz et al. 2012; Powell et al. 2013b). None of these compounds increases UGE by >50% of the estimated filtered glucose load, an observation that was difficult to explain (Liu et al. 2012) until recent studies used SGLT1 knockout (KO), SGLT2 KO, SGLT1/SGLT2 double KO (DKO), and wild-type (WT) littermate mice to show that, in the absence of SGLT2, SGLT1 can reabsorb up to 70% of the filtered glucose load under normal physiological conditions (Powell et al. 2013a). Further studies confirmed these findings and also showed that the selective SGLT2 inhibitor empagliflozin, which reduced glucose reabsorption by only 56% in WT mice, reduced it by 100% in SGLT1 KO mice (Rieg et al. 2014). These data, additional indirect evidence (Abdul-Ghani et al. 2013), and the knowledge that none of the currently available compounds should be able to inhibit renal SGLT1 at the doses administered orally in clinical trials, are consistent with the conclusion that both of these renal SGLTs must be inhibited to maximize UGE.

Although the evidence clearly suggests that renal SGLT1 and SGLT2 must both be inhibited to maximize UGE, there is no experimental evidence showing that simultaneous pharmacologic inhibition of these two renal SGLTs by an orally available agent results in UGE values approaching 100% of the estimated filtered glucose load. The studies reported here used LP-925219 [(2S,3R,4R,5S,6R)-2-(4-chloro-3-(4-methoxybenzyl)phenyl)-6-(methylthio)tetrahydro-2H-pyran-3,4,5-triol], a novel, orally available dual SGLT1/SGLT2 inhibitor with structure and preclinical pharmacokinetics very similar to that of sotagliflozin, to test whether providing such a compound to WT mice can increase UGE to the maximal value found in DKO mice.

## Materials and Methods

### Mice

All studies were performed at Lexicon Pharmaceuticals, Inc., in strict accordance with the recommendations in the Guide for the Care and Use of Laboratory Animals of the National Institutes of Health. The protocols for all studies were approved by the Lexicon Institutional Animal Care and Use Committee (OLAW Assurance Number, A4152-01; AAALAC International Accreditation Number, 001025).

General methods for mouse care have been described (Powell et al. 2013a). SGLT1 KO, SGLT2 KO, and SGLT1/SGLT2 DKO mice are published (Powell et al. 2013a). SGLT1 and SGLT2 heterozygous (HET) mice were used to generate SGLT1 HET/SGLT2 KO and SGLT1 KO/SGLT2 HET mice; all of these mice were on a C57BL/6J-129 SvEv hybrid background. In each study involving these KO lines, male mice were maintained on glucose-free diet containing 45% kcal from fat (lard) and 35% kcal from fructose (G-free diet; product # D08040105i; Research Diets, New Brunswick, NJ). Male C57BL/6-*Tyr*c-Brd (C57) mice, obtained from an in-house colony and used for pharmacokinetic studies, were fed a standard rodent chow diet (5010, LabDiet; PMI Nutrition International, St. Louis, MO) containing 23% calories as fat.

### Meal challenge

A meal consisting of low-fat diet (10% kcal as fat, D12450B; Research Diets, New Brunswick, NJ) supplemented with glucose was prepared by adding 50 g of low-fat diet powder and 9.4 g of glucose to water with a final volume of 94 mL. At 8:30 am, C57 mice in the fed state received either vehicle or 50 mg/kg LP-925219 by oral gavage; 30 min later, at Time = 0, each mouse received 25 mL/kg of the above meal (9.2 g/kg glucose, 2.5 g/kg protein, 0.6 g/kg fat) by oral gavage as described previously (Powell et al. 2013b). Blood obtained from unanesthetized mice at 0, 10, and 30 min by tail nick, and at 60 min by retro-orbital bleed, was assayed for whole blood glucose using an Accu-Chek Aviva glucometer (Roche Diagnostics, Indianapolis, IN). The blood obtained at 60 min was also used to measure circulating levels of total GLP-1 (tGLP-1; Glucagon-Like Peptide-1 Total ELISA Kit, catalog #EZGLP1T-36K, EMD Millipore, Billerica, MA) as described previously (Powell et al. 2013b). After their blood was drawn at 60 min, mice were anesthetized with isoflurane (TW Medical Veterinary Supply, Lago Vista, TX) and then euthanized by cervical dislocation. Cecal contents were then collected and analyzed for glucose and pH as described previously (Powell et al. 2013b). Blood glucose time-course data were converted to area under the curve (AUC) values by trapezoidal summation using GraphPad Prism v4.03.

### Metabolic cage studies

Mice were individually housed in Nalgene Metabolic Cages for Mice (product MTB-0311), and 24-h urine collections were made to determine 24-h UGE, as described previously (Powell et al. 2013a). Some mice received a single dose of either LP-925219 or vehicle by oral gavage at 8 am immediately prior to initiating collection of 24-h urine samples; in addition, some mice received two doses of either vehicle or 60 mg/kg LP-925219 by oral gavage, with the first dose given at 8 am and the second dose given at 6 pm, nearly midway through the collection of the 24-h urine sample.

### Glomerular filtration rate

Glomerular filtration rate (GFR) was measured in conscious mice by plasma clearance kinetics of fluorescein isothiocyanate (FITC) after a single intravenous bolus injection of FITC-inulin (Sigma, St. Louis, MO) as described previously (Powell et al. 2013a).

### LP-925219 synthesis

LP-925219 was synthesized at Lexicon Pharmaceuticals, Inc., using the method outlined in Supplemental Data.

### SGLT1 and SGLT2 cell lines

Methods for generating cell lines expressing mouse, rat, and dog SGLT1 and SGLT2 are published (Powell et al. 2013b, 2014). Full-length coding sequence of human SGLT1 (NP_000334) and human SGLT2 (NP_003032.1) with an HA-tag at the N-terminus were cloned into the mammalian expression vector pIRESpuro2 (Clontech, Mountain View, CA). HEK293 cells (ATCC, Manassas, VA) were transfected with the pIRESpuro2 vector containing human SGLT1 or SGLT2 and bulk stable cell lines were selected in the presence of 0.5 *μ*g/mL of puromycin. Cells expressing each SGLT were maintained in DMEM media containing 10% fetal bovine serum, 2 mol/L L-glutamine, 100 units penicillin/mL, 0.1 mg/mL streptomycin and 0.5 *μ*g/mL puromycin, and were used in experiments to determine compound IC_50_ (concentration causing half-maximal inhibition) values.

### α-Methylglucopyranoside uptake assay

Cell lines expressing SGLT1 or SGLT2 were plated into poly-D-lysine-coated 384-well plates, and culture overnight in cell growth medium. Cells were washed, and then incubated in uptake buffer (140 mol/L NaCl, 2 mol/L KCl, 1 mol/L CaCl_2_, 1 mol/L MgCl_2_, 10 mol/L HEPES/Tris, 1 mg/mL of bovine serum albumin, pH7.4) containing [^14^C]α-methylglucopyranoside (^14^C-AMG), a nonmetabolizable glucose analog specific for sodium-dependent glucose transporters (Wright and Turk 2004). The inhibition of SGLT1 or SGLT2 was determined by measuring SGLT1/2-mediated ^14^C-AMG uptake in the presence of increasing compound concentrations of LP-925219. Phlorizin, a well characterized, nonselective inhibitor of SGLTs was used as reference compound (Ehrenkranz et al. 2005). The percent inhibition of SGLT-mediated ^14^C-AMG uptake at different compound concentrations was calculated as follows: % Inhibition = (*B* − *X*/*B *− *A*) × 100, where *A* is the uptake in the presence of 100 *μ*mol/L phlorizin (baseline response; no SGLT-mediated uptake); *B* is the uptake in the absence of SGLT inhibitor (maximum response, total uptake); and *X* is the ^14^C-AMG uptake at a given compound concentration. Standard sigmoidal dose–response model curves were fitted, and the IC_50_ value was computed as the LP-925219 concentration that inhibited the ^14^C-AMG uptake by 50% between baseline and maximum uptake.

### Compound washout study

The uptake of ^14^C-AMG by cells expressing human SGLT1 or human SGLT2 was measured either in the presence of increasing concentrations of LP-925219 or phlorizin (no wash protocol), or determined 20 h after compound washout (washout protocol), and IC_50_ values were then calculated. Cells in the washout protocol were (1) washed; (2) incubated in uptake buffer containing increasing concentrations of LP-925219 or phlorizin at 37°C for 30 min; (3) extensively washed and then incubated in cell culture medium overnight, total recovery time 18–20 h; (4) washed again; and (5) studied in the ^14^C-AMG uptake assay. Cells in the no wash protocol were (1) washed; (2) incubated in uptake buffer in the absence of LP-925219 or phlorizin at 37°C for 30 min; (3) extensively washed and then incubated in cell culture medium overnight; (4) washed again; and (5) studied in the ^14^C-AMG uptake assay in the presence of increasing concentrations of LP-925219 or phlorizin.

### Pharmacokinetics

Adult male C57BL/6J mice (25–35 g) were maintained on chow diet, had free access to water and were conscious through the study. LP-925219 was delivered by bolus intravenous administration at 1 mg/kg or by oral gavage at a dose of 10 mg/kg; 0.1% Tween-80 served as vehicle. Following intravenous injection, serial blood samples were collected in Ethylenediaminetetraacetic acid-containing tubes through 6 h. Following oral administration, serial blood samples were collected through 24 h. The plasma fraction was immediately separated by centrifugation at 4°C, and then stored at −20°C until sample analysis. To quantify LP-925219 concentrations, plasma samples were extracted with acetonitrile-water-formic acid (80:20:0.1) containing verapamil as internal standard at a concentration of 1 *μ*mol/L. Extracted samples were analyzed by LC/MS-MS using a Surveyor HPLC system with a TSQ triple quadrupole mass spectrometer coupled with an Electrospray ionization source (Thermo Fisher Scientific, Waltham, MA). XCalibur 2.0 SR2 software (Thermo Fisher Scientific) was used for instrument control and data calculation. A rapid LC gradient with ammonium acetate 10 mol/L (pH ∼6) and acetonitrile was used. Calculations were made by normalizing the area counts of compound to the area counts of the internal standard (relative area counts).

### Pharmacokinetic data analysis

All plasma concentration versus time data for LP-925219 were analyzed using noncompartmental analysis, either extravascular administration (for oral administration) or bolus IV administration (for intravenous administration), of Phoenix™ WinNonlin® (version 6.3, Pharsight, Inc. Mountain View, CA). The half-life during the terminal phase was calculated from the elimination rate constant (*λ*) determined by the linear regression analysis of the log-linear part of the plasma concentration curve. The LP-925219 AUC_0–*t*_ was calculated using linear up/log down trapezoidal method up to the last measured concentration at time *t* (*C*_*t*_). AUC_0–∞_ was calculated as AUC_0–*t*_ + *C*_*t*_/*λ*. Clearance (CL) was calculated by dose/AUC_0–∞_. Other pharmacokinetic parameters included plasma peak concentration (*C*_max_), the time of *C*_max_ (*T*_max_) and volume of distribution at steady state (*V*_ss_). Oral bioavailability (%*F*) was calculated by the equation: %*F* = (AUC_0–∞_ PO/AUC_0–∞_ IV) × (Dose IV/Dose PO).

### Statistics

Results are presented as mean ± SD. Comparisons between two groups were analyzed by unpaired Student's *t*-test. Comparisons among three or more groups were analyzed by one-way ANOVA, with post hoc analysis performed on all comparisons using the Bonferroni correction. All statistical tests were performed using PRISM 4.03 (GraphPad). Differences were considered statistically significant when *P *<* *0.05.

## Results

**Figure 1:**
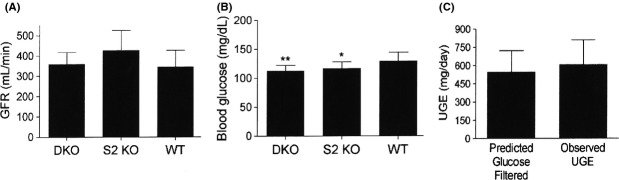
Predicted glucose filtered and observed UGE values are comparable in DKO mice. (A) GFR and (B) fed blood glucose values measured in DKO, SGLT2 (S2) KO, and wild-type (WT) littermate mice. (C) For DKO mice, predicted glucose filtered (GFR × 1440 min/day × fed blood glucose) and observed UGE values. *N* = 10–12 mice/group. Blood glucose <WT blood glucose: *P <* *0.05, **P <* *0.01. UGE, urinary glucose excretion.

In order to firmly establish that SGLT1/SGLT2 DKO mice have maximal UGE, we first measured GFR and blood glucose levels in WT, DKO and SGLT2 KO mice. As shown in Figure1A, the 3 groups of mice had comparable GFR values. As shown in Figure1B, DKO and SGLT2 KO mice had significantly lower blood glucose levels than WT littermates. For DKO mice, these data were then entered into the equation (GFR [*μ*L/min] × 1440 min/day × blood glucose [mg/dL]), which yielded a predicted value of 586 ± 126 mg of glucose filtered daily by the kidney. This predicted value was not statistically different from the UGE value of 648 ± 163 mg/day measured in these same DKO mice, suggesting that the 24-h UGE of DKO mice was at, or very close to, maximal values (Fig.1C).

**Figure 2:**
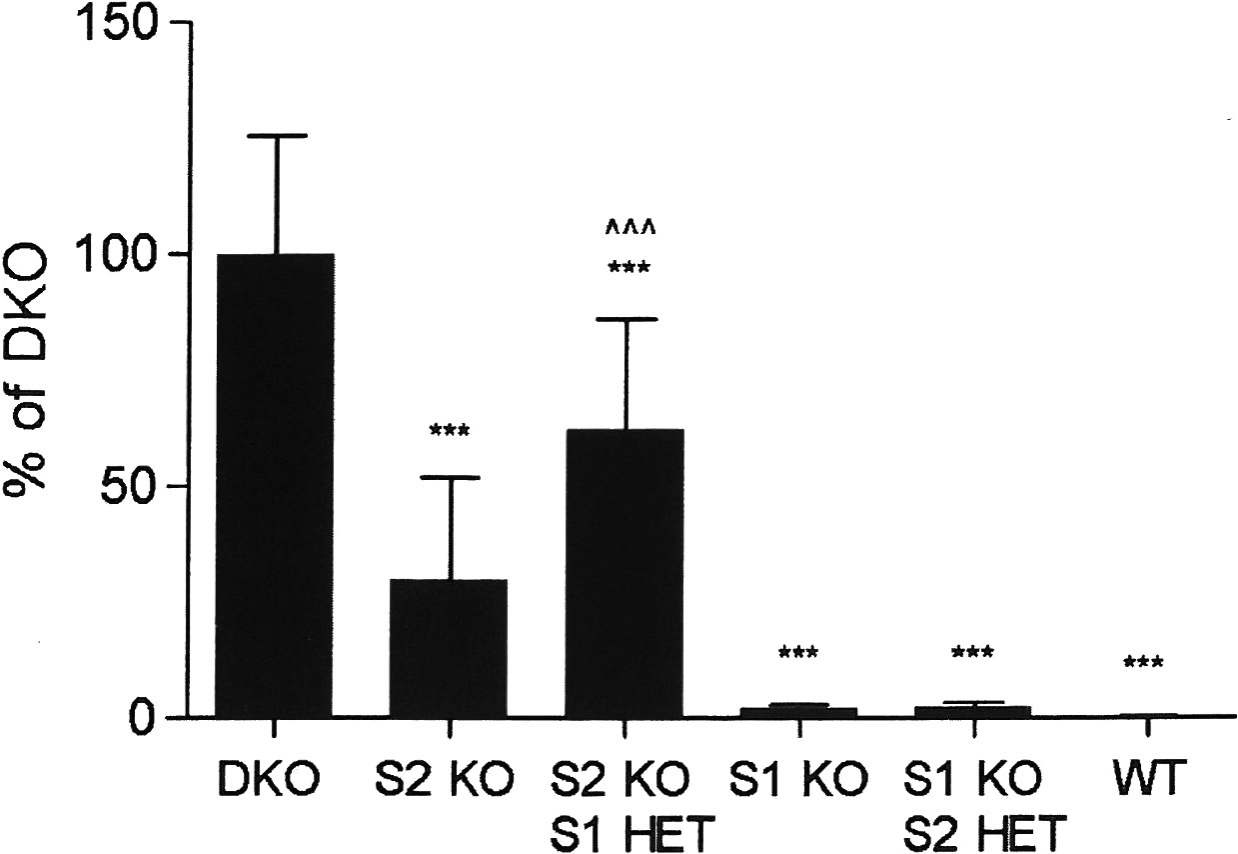
UGE values are highest in DKO mice. UGE was measured in DKO, SGLT2 (S2) KO, SGLT1 (S1) KO, S2 heterozygous (HET)/S1 KO, S1 HET/S2 KO, and WT littermate mice. *N* = 8–12 mice/group. UGE < DKO UGE, ***P <* *0.001; UGE > S2 KO UGE, ^^^*P *<* *0.001. UGE, urinary glucose excretion.

We next measured 24-h UGE under normal physiological conditions in mice with varying copy number of the SGLT1 and SGLT2 genes (Fig.2). As expected, DKO mice had the highest UGE, measuring 606 ± 154 mg/day; this was considered to be the maximal UGE and was assigned a value of 100%. In comparison to DKO mice, WT mice essentially reabsorbed all filtered glucose, and had a UGE value of only 1.0 ± 0.33 mg/day. The UGE of SGLT2 KO mice was 30 ± 22% of maximal, indicating that there was enough SGLT1 present to reabsorb 70% of filtered glucose in the absence of SGLT2. In contrast, the UGE of SGLT2 KO/SGLT1 HET mice was 62 ± 24% of maximal, indicating that the decrease in SGLT1 gene copy number was associated with a decreased reabsorption of filtered glucose. The UGE of SGLT1 KO mice was only 2 ± 1% of maximal, indicating that SGLT2 present in these mice could reabsorb 98% of filtered glucose; interestingly, the UGE of SGLT1 KO/SGLT2 HET mice was also only 2 ± 1% of maximal.

**Figure 3:**
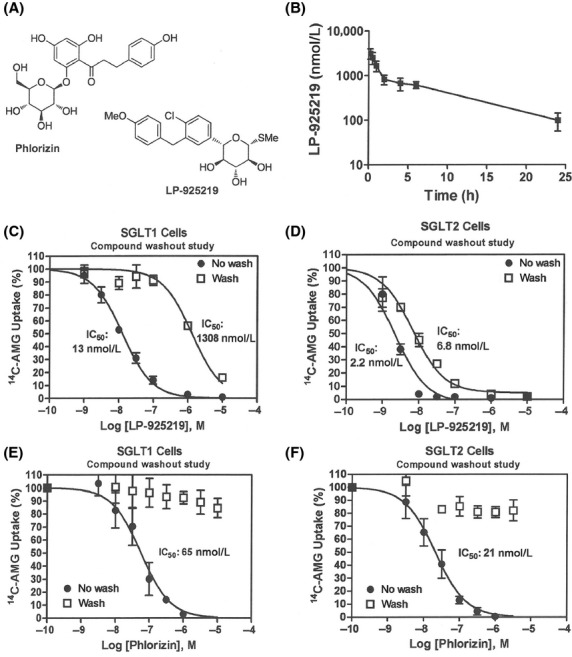
Characterization of LP-925219. (A) Left top, structure of phlorizin. Right bottom, structure of LP-925219, (2S,3R,4R,5S,6R)-2-(4-chloro-3-(4-methoxybenzyl)phenyl)-6-(methylthio)tetrahydro-2H-pyran-3,4,5-triol. (B) Plasma concentrations of LP-925219 measured at various times following an oral dose of 10 mg/kg. C) LP-925219 inhibition of human SGLT1 activity in vitro after washout followed by prolonged recovery time. After cells expressing human SGLT1 were treated with increasing concentrations of LP-925219 for 30 min at 37°C, they were extensively washed, allowed to recover for 18–20 h in cell growth medium, washed again, and then assayed for human SGLT1 activity (Washout). Control cells were incubated in the absence of LP-925219 at 37°C for 30 min, extensively washed, incubated in cell culture medium overnight, washed again, and then assayed for human SGLT1 activity in the presence of increasing concentrations of LP-925219 (No wash). (D) LP-925219 inhibition of human SGLT2 activity in vitro after washout followed by prolonged recovery time. Cells expressing human SGLT2 were studied using the above protocol. (E) Phlorizin inhibition of human SGLT1 activity in vitro after washout followed by prolonged recovery time. Cells expressing human SGLT1 were studied using the above protocol. (F) Phlorizin inhibition of human SGLT2 activity in vitro after washout followed by prolonged recovery time. Cells expressing human SGLT2 were studied using the above protocol.

We selected LP-925219 to test whether an orally available dual inhibitor of SGLT1 and SGLT2 can maximally block the reabsorption of glucose filtered by the kidney. The structure of LP-925219 is shown in Figure3A. As shown in Table1, LP-925219 inhibited mouse SGLT1 and SGLT2 with IC_50_ values of 22.6 ± 1.0 and 0.5 ± 0.1 nmol/L, respectively, and was also a potent inhibitor of rat, dog, and human SGLT1 and SGLT2 in vitro. The major pharmacokinetic parameters of LP-925219 after intravenous or oral administration to mice are shown in Table2 and 3, respectively, and the plasma concentration-time profile after a 10 mg/kg oral dose is presented in Figure3B. Following intravenous administration, the half-life of LP-925219 was 2.8 h and the CL was 29 mL/min per kg. Following oral administration of a 10 mg/kg dose, LP-925219 had a *T*_max_ of 0.3 h and a half-life of 7 h, and was well absorbed with a bioavailability (%*F*) of 87%; LP-925219 plasma levels remained high at 6 h postdosing (617 ± 107 nmol/L), and were still 100 ± 43 nmol/L at 24 h post dosing. We also performed in vitro compound washout experiments in an attempt to assess the effective residence time of LP-925219 at SGLT1 (Fig.3C) and SGLT2 (Fig.3D), and compared these results to the residence time of phlorizin at SGLT1 (Fig.3E) and SGLT2 (Fig.3F). We found that LP-925219 displayed a more prolonged residence time with cells expressing SGLT2 compared to cells expressing SGLT1; in contrast, phlorizin had a much shorter residence time, and was unable to significantly inhibit ^14^C-AMG transport by either SGLT1 or SGLT2 20 h after phlorizin washout.

**Table 1:** LP-925219 inhibits SGLT-mediated glucose transport.

Species	SGLT1 IC50 (nmol/L)	*N*	SGLT2 IC50 (nmol/L)	*N*
Mouse	22.6 ± 1.0	5	0.5 ± 0.1	4
Rat	36.7 ± 7.2	3	0.3 ± 0.2	3
Dog	30.2 ± 2.0	5	1.0 ± 0.3	4
Human	15.9 ± 4.2	11	2.1 ± 0.5	11

Data are mean ± SD; *N*, number of determinations. SGLT2, sodium/glucose cotransporter 1.

**Table 2:** Pharmacokinetic parameters after a 1 mg/kg intravenous dose of LP-925219.

Half-life (h)	*C*_max_ (nmol/L)	AUC_0-*t*_ (nmol/L × h)	AUC_0-∞_ (nmol/L × h)	CL (mL/min per kg)	Vss (L/kg)
2.8 ± 0.7^	1436 ± 97^1^	1214 ± 12^1^	1409 ± 40^	29 ± 1^	4.4 ± 0.6^

Data are mean ± SD; *C*_max_, plasma peak concentration; AUC_0-*t*_, AUC from time 0 to time of last measured concentration at time *t* (*C*_*t*_); AUC_0-*∞*_, AUC_0-*t*_ + Ct/*λ*; CL, clearance; Vss, volume of distribution at steady state.

1 *N* = 4; ^*N *= 3.

**Table 3:** Pharmacokinetic parameters after a 10 mg/kg oral dose of LP-925219.

Half-life (h)	*T*_max_ (h)	*C*_max_ (nmol/L)	AUC_0–*t*_ (nmol/L × h)	AUC_0-*∞*_ (nmo/L*h)	%F (h)
7.0 ± 0.9	0.3 ± 0	3164 ± 802	11,164 ± 2,507	12,214 ± 3037	87 ± 21

Data are mean ± SD; *N* = 4 for each parameter; *T*_max_, time of plasma peak concentration; *C*_max_, plasma peak concentration; AUC_0-*t*_, AUC from time 0 to time of last measured concentration at time *t* (*C*_*t*_); AUC_0-*∞*_, AUC_0-*t*_ + *C*_*t*_/*λ*; %F, bioavailability.

**Figure 4:**
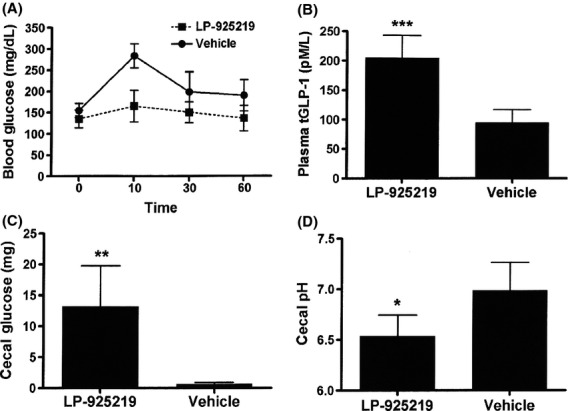
LP925219-treated mice respond to a glucose-containing meal challenge with increased levels of plasma GLP-1 and cecal glucose, and decreased levels of blood glucose and cecal pH. Mice received either vehicle or LP-925219 at a dose of 50 mg/kg (*n* = 5 for each group) by oral gavage; 30 min later the mice received, by oral gavage at Time 0, a glucose-containing meal challenge. (A) Blood glucose levels were measured at 0, 10, 30, and 60 min; for each mouse, blood glucose levels were used to calculate an AUC value for statistical analysis. At 60 min, blood was obtained for (B) tGLP-1 levels, and cecal contents were obtained and assayed for (C) cecal glucose and (D) cecal pH. LP-925219-treated value different from vehicle, *P <* *0.05, **P <* *0.01, ***P <* *0.001. AUC, area under the curve.

The ability of LP-925219 to inhibit intestinal SGLT1 in vivo was demonstrated by studies presented in Figure4. Mice that received 50 mg/kg of LP-925219 by oral gavage responded to a glucose-containing meal challenge with a significant decrease in blood glucose AUC (*P *<* *0.05; Fig.4A) and increased levels of circulating total GLP-1 (Fig.1B). In addition, their cecal contents contained more glucose, and had a lower pH, than did the cecal contents of mice that received vehicle by oral gavage (Fig.4C–D); diarrhea was not observed in any mouse during this short-term study. These results are consistent with LP-925219-mediated inhibition of SGLT1-mediated uptake of dietary glucose by the small intestine.

**Figure 5:**
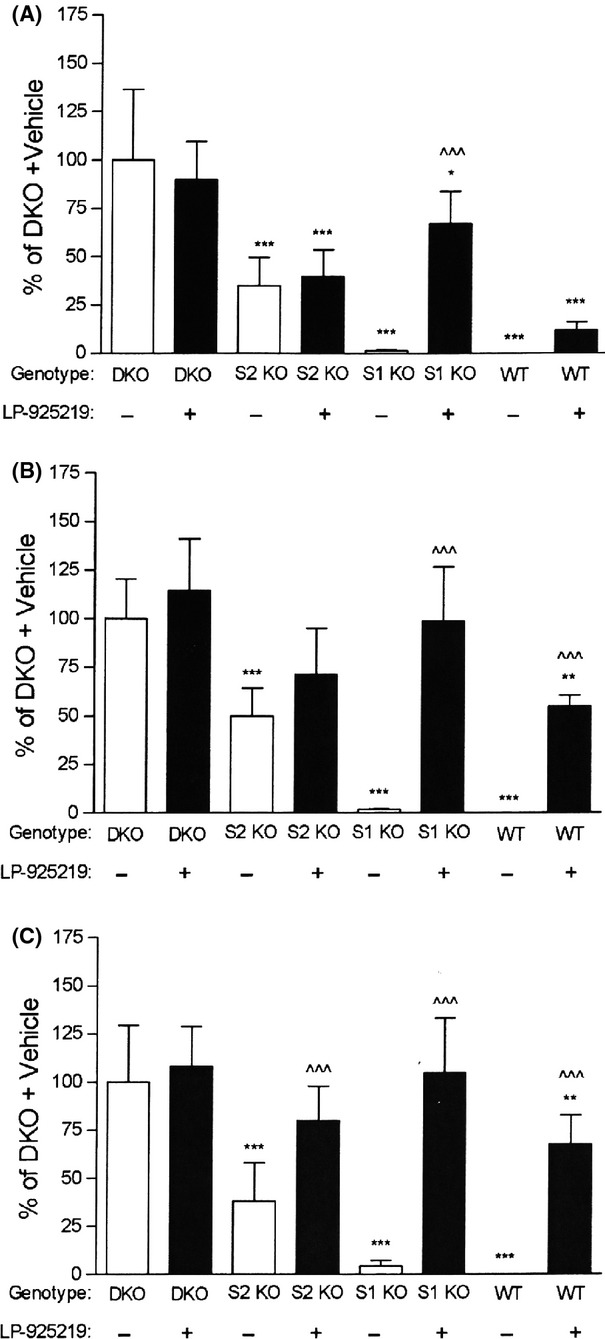
UGE values in mice treated with a single dose of LP-925219. After DKO, S2 KO, S1 KO, and WT littermate mice received, by a single oral gavage at 8 am, either vehicle (Veh) or LP-925219 at a dose of (A) 1 mg/kg; (B) 10 mg/kg; or (C) 60 mg/kg, the mice were placed in metabolic cages and their urine was collected over the next 24 h and analyzed for total glucose. *N* = 5–11 mice/group. UGE < DKO UGE: *P <* *0.05, **P <* *0.01, ***P <* *0.001; LP-925219 UGE > Veh UGE for the same group, ^^^*P *<* *0.001. UGE, urinary glucose excretion.

We next tested the ability of single 1, 10, or 60 mg/kg doses of LP-925219 to inhibit reabsorption of filtered glucose by the renal tubules (Fig.5). The 24-h UGE of DKO mice was not significantly increased by any dose of LP-925219. This is consistent with the UGE of DKO mice being a maximal value, and is the reason why the 24-h UGE of vehicle-treated DKO mice was assigned a value of 100% (maximal) for each study. The 24-h UGE of SGLT1 KO mice responded to LP-925219 with a dose-dependent increase that was significantly greater than vehicle-treated values and almost maximal at 1 mg/kg, and was maximal at all higher doses. In contrast, the 24-h UGE of SGLT2 KO mice did not show a significant increase with the 1 or 10 mg/kg doses of LP-925219, and required a 60 mg/kg dose to reach a UGE which was significantly greater than vehicle-treated controls and, at 80 ± 18% of maximal, was not significantly different from maximal values. Further, the 24-h UGE of WT mice did not show a significant response to the 1 mg/kg dose of LP-925219, and the response to the 60 mg/kg dose, which was 68 ± 15% of maximal, was significantly greater than vehicle-treated control values but was still significantly less than maximal values.

**Figure 6:**
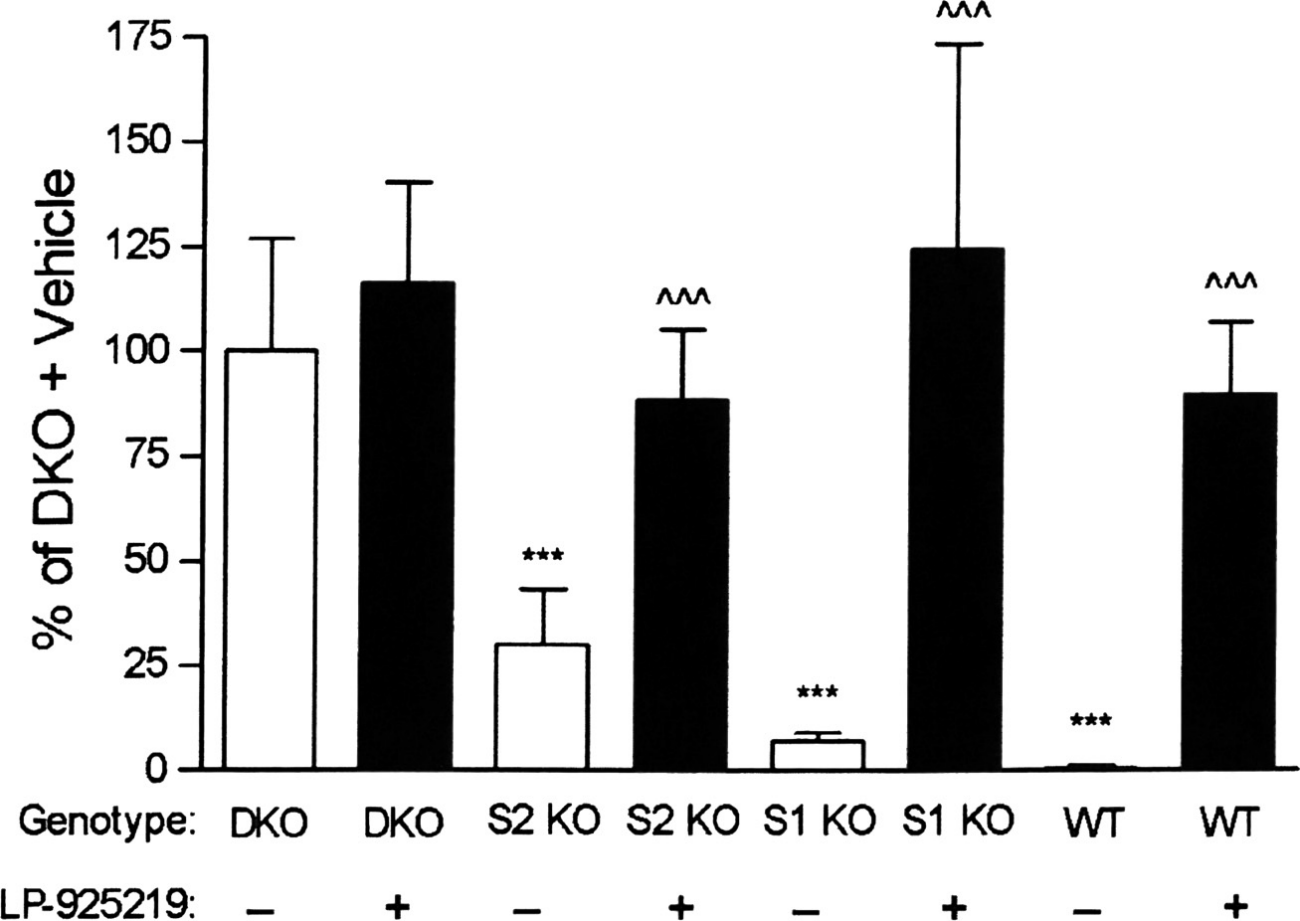
UGE values in mice treated with two doses of 60 mg/kg LP-925219. DKO, S2 KO, S1 KO, and WT littermate mice received, by oral gavage at 8 am and then again at 6 pm, either vehicle (Veh) or LP-925219 at a dose of 60 mg/kg. The mice were placed in metabolic cages after the first gavage and their urine was collected over the next 24 h and analyzed for total glucose. *N* = 6–11 mice/group. UGE < DKO UGE, ***P <* *0.001; LP-925219 UGE > Veh UGE for the same group, ^^^*P *<* *0.001. UGE, urinary glucose excretion.

We hypothesized that LP-925219 might be unable to maximally inhibit SGLT1 in SGLT2 KO and WT mice over 24 h because LP-925219 has a much higher IC_50_, and a much lower residence time, for SGLT1 compared to SGLT2. For these reasons, we next delivered two 60 mg/kg doses of LP-925219 by oral gavage, with the first dose given at 8 am and the second dose given at 6 pm, to the same mice. As is shown in Figure6, the 24-h UGE was again not significantly increased by LP-925219 in DKO mice but was maximally increased in SGLT1 KO mice. In addition, under this dosing regimen, the 24-h UGE values of SGLT2 KO and WT mice were not different from maximal, measuring 89 ± 16% (*P *>* *0.05) and 90 ± 17% (*P *>* *0.05), respectively, of maximal values.

## Discussion

These studies demonstrate that oral delivery of LP-925219 can maximize UGE by inhibiting both renal SGLT1 and SGLT2. This conclusion was based on the use of 24-h UGE values from DKO mice as maximal values, which was predicated on the hypothesis that DKO mice do not reabsorb filtered glucose. We provided evidence supporting this hypothesis by measuring GFR and blood glucose levels to estimate the amount of glucose/day filtered in DKO mice, and then showing that this value was comparable to 24-h UGE values obtained from the same mice on the following day. These results confirm past studies that compared the estimated amount of glucose filtered/day in one DKO cohort with 24-h UGE values from an independent cohort (Powell et al. 2013a), and are consistent with CL studies showing the absence of glucose reabsorption in DKO mice (Rieg et al. 2014). It is important to point out that the DKO mice appear quite healthy. In past studies, there were no clinically significant differences among DKO, SGLT1 KO, SGLT2 KO, and WT littermates for any blood chemistries; also, although DKO mice had increased urine volume due to a glucose-induced osmotic diuresis, it was offset by greater water intake, and although they showed increases in some urine electrolytes, these increases were not a problem as they were simply the result of greater food intake (Powell et al. 2013a; Rieg et al. 2014).

Before performing studies with LP-925219, we measured 24-h UGE in mice with varying copy number of the SGLT1 and SGLT2 genes in order to better understand the effect of titrating these genes on glucose reabsorption and UGE under normal physiological conditions. As expected, DKO mice had the highest 24-h UGE which was assigned the maximal value of 100%, and WT mice had the lowest value which was <0.2% of maximal. SGLT2 KO mice had a value that was 30% of maximal, consistent with past results showing that mice with two copies of the SGLT1 gene can reabsorb 70% of filtered glucose in the absence of SGLT2 under normal physiological conditions (Powell et al. 2013a). Interestingly, the UGE of SGLT2 KO/SGLT1 HET mice was 62% of maximal, indicating that these mice, which have only ½ the SGLT1 gene copy number present in SGLT2 KO mice, could reabsorb only 38% of filtered glucose, roughly half the amount reabsorbed by SGLT2 KO mice. Unfortunately, our attempts to verify SGLT1 protein titration using a variety of anti-SGLT1 antibodies were unsuccessful due to the lack of specificity shown by each antibody during validation studies performed by western blot (data not shown). Nevertheless, our results strongly suggest that SGLT1 gene titration directly affects the abundance of SGLT1 transporters, and that significant compensation does not occur, consistent with the lack of compensation during the titration of many other genes (Smithies et al. 2000). One clinically relevant implication of this finding is that individuals with a single functional SGLT1 gene may respond to complete pharmacological inhibition of SGLT2 with twice the usual 24-h UGE; indeed, SGLT1 heterozygosity should be considered as a possible explanation for the extremely high UGE values in one SGLT2-deficient individual (Oemar et al. 1987; Scholl-Burgi et al. 2004). The 24-h UGE of SGLT1 KO mice was 2% of maximal, consistent with past results suggesting the renal tubular SGLT2 present in these mice reabsorbs 97–98% of filtered glucose (Powell et al. 2013a; Rieg et al. 2014). Interestingly, the 24-h UGE of SGLT1 KO/SGLT2 HET mice was also 2% of maximal; assuming the likely absence of compensation during SGLT2 gene titration, this finding indicates that there are at least twofold more SGLT2 transporters present in the renal tubules of WT and SGLT1 KO mice than are needed to completely reabsorb the filtered glucose load under normal physiological conditions.

LP-925219 was selected for an attempt to maximally inhibit renal glucose reabsorption for many reasons: (1) in vitro, it was more potent than sotagliflozin as an inhibitor of both mouse SGLT1 and mouse SGLT2 (Powell et al. 2013b). (2) During washout studies, LP-925219 showed a prolonged ability to inhibit SGLT2 and, to a lesser extent, SGLT1. This prolonged inhibition may be due to a slow dissociation rate of LP-925219 from these SGLTs; such an explanation is consistent with the inability of phlorizin, which rapidly dissociates from SGLT1 and SGLT2 (Hummel et al. 2012), to inhibit either of these SGLTs under the same washout conditions. The prolonged inhibition may also be due to intracellular accumulation of LP-925219 where it can still inhibit these transporters, but recent data suggest that SGLT2 inhibitors act only on the extracellular surface (Ghezzi et al. 2014), making this explanation less likely. Whatever the mechanism, it is clear that prolonged occupancy of a drug at its target can provide a long-lasting pharmacodynamic effect that outlasts the pharmacokinetics (Swinney 2008, 2009; Tummino and Copeland 2008; Liu et al. 2012). (3) In vivo, orally administered LP-925219 was highly bioavailable with a prolonged plasma half-life and with an overall pharmacokinetic profile in mice that was quite similar to that of sotagliflozin (Powell et al. 2014). (4) LP-925219 clearly inhibited SGLT1-mediated absorption of dietary glucose in vivo as evidenced by decreased blood glucose excursions, increased cecal glucose, decreased cecal pH, and increased circulating GLP-1 after a glucose-containing meal, a pattern reminiscent of that observed in SGLT1 KO mice and in WT mice treated with sotagliflozin (Powell et al. 2013b).

The ability of LP-925219 to inhibit renal glucose reabsorption was initially studied by providing a single LP-925219 dose of 1, 10, or 60 mg/kg delivered by oral gavage. None of these doses significantly increased UGE in DKO mice. In SGLT1 KO mice, LP-925219 increased UGE in a dose-dependent manner with maximal effect achieved at a dose of 10 mg/kg; this suggests that the large amount of SGLT2 present in the tubules of SGLT1 KO mice was easily inhibited by LP-925219, consistent with clinical studies using other SGLT2 inhibitors (Musso et al. 2011) and confirming results from a previous study that used the selective SGLT2 inhibitor empagliflozin to completely inhibit glucose reabsorption in SGLT1 KO mice (Rieg et al. 2014). Single doses of LP-925219 also increased UGE in SGLT2 KO and WT mice in a dose-dependent manner, but the increases were less robust than those observed in SGLT1 KO mice and in fact were submaximal at all doses in WT mice. This failure of LP-925219 to robustly inhibit renal SGLT1 in WT and SGLT2 KO mice was unlikely to result from proximal reabsorption of the compound upstream of SGLT1, because the most likely proximal transporter, SGLT2, is absent in SGLT2 KO mice. More likely explanations relate to the pharmacokinetics of LP-925219: (1) LP-925219 has a much higher IC_50_ for mouse SGLT1 than mouse SGLT2, which becomes a problem if the compound is highly bound by plasma proteins. Although plasma protein binding was not directly measured for LP-925219, it is likely to be quite high because sotagliflozin, a compound with very similar structure and pharmacokinetic properties, has plasma protein binding of >90% in mouse and human (Powell et al. 2014). Compounds with similar time-course PK profiles, such as LP-925219, sotagliflozin and selective SGLT2 inhibitors, would be expected to have similar plasma protein binding, and indeed there are no published reports describing major differences in plasma protein binding among the SGLT2 inhibitors currently marketed or in clinical development (Liu et al. 2012). If this is the case, levels of free LP-925219 are unlikely to be high enough to maximally inhibit renal SGLT1 over the entire 24 h of urine collection, because LP-925219 levels at 24 h were 100 nmol/L, which is only 4 times higher than the IC_50_ for SGLT1. (2) LP-925219 is more easily washed off of cells expressing SGLT1 than cells expressing SGLT2. Although the relevance of this observation to the in vivo effects of LP-925219 is not clear, it may contribute to the inability of LP-925219 to maximally inhibit glucose reabsorption by renal tubular SGLT1 over a period of 24 h.

Based on the above findings, the same mice received two 60 mg/kg doses of LP-925219, the first given at 8 am and the second given at 6 pm, in an attempt to raise compound levels during the last 12 h of urine collection. With this protocol, LP-925219 now maximally increased 24-h UGE in WT and SGLT2 KO mice, in addition to SGLT1 KO mice. Thus, an orally available compound can indeed maximize 24-h UGE in mammals. The results also suggest that novel dual SGLT1/SGLT2 inhibitors with lower IC_50_ for SGLT1, prolonged SGLT1 retention time and/or additional optimization of pharmacokinetic properties may be able to maximally inhibit renal glucose reabsorption with once daily oral dosing. The effect of these compounds on intestinal glucose absorption will have to be monitored closely in future studies, due to the potential advantage of partial SGLT1 inhibition leading to delayed glucose absorption and improved glycemic control, and to the potential disadvantage of excessive SGLT1 inhibition leading to frank malabsorption. Gastrointestinal effects were not evaluated in the current study because the mice were fed a glucose-free diet due to our focus on the renal effects of LP-925219. Also, the possibility that the benefits of increased UGE may be offset by increased endogenous glucose production, as occurred during oral administration of SGLT2 inhibitors (Ferrannini et al. 2014; Merovci et al. 2014), must also be evaluated. Despite these potential concerns, the fact that DKO mice had lower fasting and fed blood glucose levels throughout the day relative to SGLT2 KO littermate mice (Powell et al. 2013a) suggests that dual SGLT1/SGLT2 inhibitors merit further evaluation for their potential, in patients with diabetes, to achieve significantly better glycemic control than is currently achieved using selective SGLT2 inhibitors.

## Abbreviations

WT: wild type
%*F*: oral bioavailability
AMG: *α*-methylglucopyranoside
AUC_0–∞_: AUC_0–*t*_ + *C*_*t*_/*λ*
AUC_0–*t*_: AUC from time 0 to time of last measured concentration at time *t* (*C*_*t*_).
AUC: area under the curve
CL: clearance
*C*_max_: plasma peak concentration
*C*_*t*_: time of the last measured concentration
DKO: double knockout
GFR: glomerular filtration rate
HET: heterozygous
IC_50_: concentration causing half-maximal inhibition
KO: knockout
LC/MS: liquid chromatography/mass spectroscopy
LP-925219: (2S,3R,4R,5S,6R)-2-(4-chloro-3-(4-methoxybenzyl)phenyl)-6 (methylthio)tetrahydro-2H-pyran-3,4,5-triol
SGLT1: sodium/glucose cotransporter 1
SGLT2: sodium/glucose cotransporter 2
T2DM: type 2 diabetes mellitus
tGLP-1: total glucagon-like peptide 1
*T*_max_: time of plasma peak concentration
UGE: urinary glucose excretion
*V*_ss_: volume of distribution at steady state
*λ*: elimination rate constant

## Supporting Information

**Figure S1.** Individual steps in the synthesis of LP-925219.
