# Supplementary material for: LP-925219 maximizes urinary glucose excretion in mice by inhibiting both renal SGLT1 and SGLT2
Source: Pharmacol Res Perspect. 2015 Mar 31;3(2):e00129. doi: 10.1002/prp2.129 (PMC4448982; doi:10.1002/prp2.129)
Supplement: Supplementary file 1 — Figure S1. Individual steps in the synthesis of LP-925219. [file prp20003-e00129-sd1.docx]

**Journal of Pharmacology and Experimental Therapeutics**

**Supplemental Data for “LP-925219 Maximizes Urinary Glucose Excretion in Mice by Inhibiting Both Renal SGLT1 and SGLT2”.** David R. Powell, Melinda G. Smith, Deon D. Doree, Angela L. Harris, Wendy W. Xiong, Faika Mseeh, Alan Wilson, Suma Gopinathan, Damaris Diaz, Nicole C. Goodwin, Bryce Harrison, Eric Strobel, David B. Rawlins, Ken Carson, Brian Zambrowicz, Zhi-Ming Ding

**Method for synthesizing LP-925219 [(2S,3R,4R,5S,6R)-2-(4-chloro-3-(4-methoxybenzyl)phenyl)-6-(methylthio)tetrahydro-2H-pyran-3,4,5-triol]**

**Figure 1. Individual steps in the synthesis of LP-925219.**

**Preparation of 1-chloro-4-iodo-2-(4-methoxybenzyl)benzene (2)**

Under nitrogen, 4-iodo-1-chloro-2-(4-ethoxybenzyl)benzene^[[1]](#footnote-1)^ (11.1 g, 31mmol) was taken up into 80 mL CH_2_Cl_2_ and cooled to 0° C. BBr_3_ (40 mL, 1M in THF) was added slowly and the reaction was stirred at 0° C for two hours. The reaction was poured into saturated potassium carbonate cooled to 0° C. The reaction was stirred for 20 minutes. The solution was acidified to pH=1 with 6*N* HCl and extracted two times with CH_2_Cl_2_2. The organic layer was concentrated and the residue was purified by silica gel column chromatography to afford the desired phenol (6.44 g, 69% yield). ^1^H NMR (400 MHz, CHLOROFORM-*d*) δ ppm 3.98 (s, 2 H) 4.67 (s, 1 H) 6.80 (d, *J*=8.59 Hz, 2 H) 6.99 - 7.18 (m, 3 H) 7.41 - 7.56 (m, 2 H). MS (ES-) [M-H]^-^ = 343.

Under nitrogen, 4-(5-iodo-2-chlorobenzyl)phenol (500 mg, 1.45 mmol), methyl iodide (0.14 mL, 2.18 mmol), and potassium carbonate (400 mg, 2.9 mmol) were taken up into 10 mL acetone and heated at 50° C overnight. The reaction was cooled to room temperature and quenched with 100 mL of water and extracted two times with ethyl acetate. The organic layer was washed with brine and concentrated and the residue was purified by silica gel column chromatography (454 mg, 86% yield). ^1^H NMR (400 MHz, CHLOROFORM-*d*) δ ppm 3.82 (s, 3 H) 3.99 (s, 2 H) 6.87 (d, *J*=8.60 Hz, 2 H) 7.11 (s, 3 H) 7.47 (s, 2 H).

**Preparation of (4-chloro-3-(4-methoxybenzyl)phenyl)((3aS,5R,6S,6aS)-6-hydroxy-2,2-dimethyltetrahydrofuro [2,3-d][1,3]dioxol-5-yl)methanone (4)**

To a solution of 1-chloro-2-(4-ethoxybenzyl)-4-iodobenzene (768 mg, 2.14 mmol) in THF (5.0 mL) was added *i*-PrMgCl (2.0M in THF, 1.14 mL, 2.34 mmol) at 0-5° C., and the mixture was stirred for 0.5 h at 0-5° C. To a solution of (3aS,5R,6S,6aS)-6-hydroxy-2,2-dimethyltetrahydrofuro[2,3-d][1,3]dioxol-5-yl)(morpholino)methanone1 (**3**, 532 mg, 1.95 mmol) in THF (1.0 mL) was added t-BuMgCl (1.0M in THF, 2.11 mL, 2.11 mmol) at 0-5° C. The Grignard solution was added to the morpholino amide solution and the reaction was stirred for one hour. The reaction was quenched with saturated aq NH_4_Cl, extracted with ethyl acetate, and washed with brine. The organic layer was concentrated and the residue was purified by silica gel column chromatography to afford the desired ketone 4 (442 mg, 58%) as a white solid. ^1^H NMR (400 MHz, CHLOROFORM-*d*) δ ppm 1.37 (s, 3 H) 1.54 (s, 3 H) 2.87 - 2.93 (m, 1 H) 3.81 (s, 3 H) 4.05 - 4.16 (m, 2 H) 4.54 - 4.57 (m, 1 H) 4.57 - 4.61 (m, 1 H) 5.18 - 5.24 (m, 1 H) 6.07 (d, *J*=3.54 Hz, 1 H) 6.87 (d, *J*=8.84 Hz, 2 H) 7.13 (d, *J*=8.59 Hz, 2 H) 7.50 (d, *J*=8.34 Hz, 1 H) 7.81 (d, *J*=2.02 Hz, 1 H) 7.88 (dd, *J*=8.34, 2.02 Hz, 1 H) 7.84 - 7.91 (m, 1 H). MS (ES+) [M+H]^+^ = 419.

**Preparation of (3aS,5S,6R,6aS)-5-((S)-(4-chloro-3-(4-methoxybenzyl)phenyl) (hydroxy)methyl)-2,2-dimethyltetrahydrofuro[2,3-d][1,3]dioxol-6-ol (5)**

To a solution of (4-chloro-3-(4-methoxybenzyl)phenyl)((3aS,5R,6S,6aS)-6-hydroxy-2,2-dimethyltetrahydrofuro[2,3-d][1,3]dioxol-5-yl)methanone (1.57 g, 3.76 mol) in MeOH (80 mL) was added CeCl_3_.7H_2_O (1.7 g, 1.2 equiv) and the mixture was stirred at 20° C. for 15 minutes. NaBH_4_ (57 mg, 1.5 mmol) in 0.10 mL of 1N NaOH was added and the reaction stirred at room temperature for one hour. The reaction was quenched with saturated aq NH_4_Cl (100 mL). The mixture was concentrated under vacuum to remove MeOH and then extracted with EtOAc and washed with brine. The combined organics were concentrated under vacuum to afford the desired alcohol as a colorless oil (1.57 g, 100% yield). MS (ES+) [M+NH_4_]^+^ = 438.

**Preparation of (3S,4R,5S,6S)-6-(4-chloro-3-(4-methoxybenzyl)phenyl)tetrahydro-2H-pyran-2,3,4,5-tetrayl tetraacetate (6)**

To (3aS,5S,6R,6aS)-5-((S)-(4-chloro-3-(4-methoxybenzyl)phenyl)(hydroxy)methyl)-2,2-dimethyltetrahydrofuro[2,3-d][1,3]dioxol-6-ol (1.57 g, 3.76 mmol) was added AcOH (8.8 mL) and H_2_O (6.2 mL) and the mixture was heated to 100° C. and stirred for 15 hours. The mixture was then cooled to room temperature (20° C.) and concentrated under vacuum to give a yellow oil (crude, 1.6 g, 100% yield). MS (ES+) [M+NH_4_]^+^ = 398.

This oil was azeotroped twice with toluene. To this crude oil in 15 mL acetonitrile was added triethyl amine (3.11 mL, 22.6 mmol). Then, Ac_2_O (2.12 mL, 22.6 mmol) and DMAP (23 mg, 0.2 mmol) was added and the mixture stirred at room temperature for 4 hours. The reaction was diluted with EtOAc and washed with sat. sodium bicarbonate followed by sodium bisulfate. The organic layer was then washed with brine and dried over sodium sulfate. The organic layer was concentrated to afford the desired tetraacetate intermediate as a yellow foam (1.88 g, 91% yield). MS (ES+) [M+NH_4_]^+^ = 566.

**Preparation of (2S,3S,4R,5S,6R)-2-(4-chloro-3-(4-methoxybenzyl)phenyl)-6-(methylthio)tetrahydro-2H-pyran-3,4,5-triyl triacetate (7)**

To a solution of tetraacetate **6** (1.88 g, 3.4 mmol assuming pure) and thiourea (522 mg, 2.0 equiv) in dioxane (8 mL) was added trimethylsilyl trifluoromethanesulfonate (TMSOTf) (0.95 mL, 1.5 equiv) and the reaction mixture was heated to 80° C. for 3.5 hours. The mixture was cooled to 20° C. and MeI (0.53 mL, 2.5 equiv) and *N,N*-diisopropylethylamine (3.0 mL, 5.0 equiv) was added and the mixture was stirred at 20° C. for 15 h. The mixture was then diluted with ethyl acetate (100 mL) and washed with H_2_O and brine. The organic layer was separated and concentrated under vacuum to give a yellow solid (1.8 g, 100% yield). MS (ES+) [M+NH_4_]^+^ = 554.

**Preparation of (2S,3R,4R,5S,6R)-2-(4-chloro-3-(4-methoxybenzyl)phenyl)-6-(methylthio)tetrahydro-2H-pyran-3,4,5-triol (8)**

To a slurry of 2S,3S,4R,5S,6R)-2-(4-chloro-3-(4-methoxybenzyl)phenyl)-6-(methylthio)tetrahydro-2H-pyran-3,4,5-triyl triacetate (1.8 g, 3.4 mmol) in MeOH (20 mL) was added NaOMe in MeOH (25 wt %, 0.5 mL) at room temperature and the mixture was stirred for 2 hours. The mixture was then concentrated and purified by prep HPLC (30 x 250mm C18 column, 5–95% acetonitrile:water (10 mM ammonium acetate), 15 min, 45 mL/min) to afford the desired methyl thiolate (0.486 g, 35% yield). ^1^H NMR (400 MHz, DMSO-*d*_6_) δ ppm 2.03 (s, 3 H) 3.09 - 3.28 (m, 3 H) 3.71 (s, 3 H) 3.99 (s, 2 H) 4.09 (d, *J*=9.60 Hz, 1 H) 4.34 (d, *J*=9.60 Hz, 1 H) 4.94 (d, *J*=5.56 Hz, 1 H) 5.12 (d, *J*=4.55 Hz, 1 H) 5.20 (d, *J*=5.56 Hz, 1 H) 6.85 (d, *J*=8.59 Hz, 2 H) 7.12 (d, *J*=8.34 Hz, 2 H) 7.21 (dd, *J*=8.08, 1.77 Hz, 1 H) 7.27 (d, *J*=1.77 Hz, 1 H) 7.39 (d, *J*=8.34 Hz, 1 H). MS (ES+) [M+NH_4_]^+^ = 428.

1. Goodwin, N.C.; Harrison, B.A.; Iimura, S.; Mabon, R.; Song, Q.; Wu, W.; Yan, J.; Zhang, H.; Zhao, M. PCT Int. WO2009014970, 2009. [↑](#footnote-ref-1)
